# The Twenty-Year Story of a Plant-Based Vaccine Against Hepatitis B: Stagnation or Promising Prospects?

**DOI:** 10.3390/ijms14011978

**Published:** 2013-01-21

**Authors:** Tomasz Pniewski

**Affiliations:** Institute of Plant Genetics Polish Academy of Sciences, Strzeszyńska 34, Poznań 60-479, Poland; E-Mail: tpni@igr.poznan.pl; Tel.: +48-61-6550251; Fax: +48-61-6550301

**Keywords:** HBV antigens, hepatitis B, injection vaccines, oral vaccines, plant-based vaccines, purification, tissue processing, transient expression, transgenic plants, VLPs

## Abstract

Hepatitis B persists as a common human disease despite effective vaccines having been employed for almost 30 years. Plants were considered as alternative sources of vaccines, to be mainly orally administered. Despite 20-year attempts, no real anti-HBV plant-based vaccine has been developed. Immunization trials, based on ingestion of raw plant tissue and conjugated with injection or exclusively oral administration of lyophilized tissue, were either impractical or insufficient due to oral tolerance acquisition. Plant-produced purified HBV antigens were highly immunogenic when injected, but their yields were initially insufficient for practical purposes. However, knowledge and technology have progressed, hence new plant-derived anti-HBV vaccines can be proposed today. All HBV antigens can be efficiently produced in stable or transient expression systems. Processing of injection vaccines has been developed and needs only to be successfully completed. Purified antigens can be used for injection in an equivalent manner to the present commercial vaccines. Although oral vaccines require improvement, plant tissue, lyophilized or extracted and converted into tablets, *etc.*, may serve as a boosting vaccine. Preliminary data indicate also that both vaccines can be combined in an effective parenteral-oral immunization procedure. A partial substitution of injection vaccines with oral formulations still offers good prospects for economically viable and efficacious anti-HBV plant-based vaccines.

## 1. Premises of Plant-Based Vaccines

Hepatitis B virus (HBV), as an etiology of acute and chronic hepatitis (HepB), cirrhosis and hepatocellular carcinoma (HCC), was identified in the 1960s [1]. The first licensed anti-HBV vaccine appeared after almost 20 years [2]. This first-generation vaccine contained subviral particles (SVPs) of HBV purified from the inactivated serum of carriers. The vaccine revealed very high efficacy [3], but its quantities soon proved insufficient and their production costly. Introduction of a subunit vaccine founded on a basic HBV immunogen, *i.e.*, small surface antigen (S-HBsAg) in the early 1980s [4] was an unquestionable success in HepB prevention. Manufacturing of the recombinant antigen (rHBsAg) in yeast met safety expectations and also made derived vaccines much cheaper than first-generation vaccines. Virus-like particles (VLPs) assembled of recombinant S-HBsAg were almost identical and as immunogenic as natural SVPs, hence vaccines containing rHBsAg (Engerix B^®^, Recombivax^®^, Euvax, *etc.*) exhibited one of the highest effectiveness rates (90%–95%). However, the initial price, *ca.* $40 per single dose, as well as the necessity of three intramuscular injections, cold chain distribution, and the accompanying infrastructure, still constituted an economical barrier for the most needy in developing countries. Moreover, it eventually turned out that some groups of people, especially the elderly, the obese, and patients with immunodeficiency syndromes in addition to other disorders, did not react sufficiently to subunit vaccines based on S-HBsAg [5,6]. A more effective therapy of chronic HepB was also increasingly necessitated in a view of expensive, often insufficient and side-effect-inducing treatments using interferons or nucleoside analogs [7,8]. Hence, since the late 1980s, many research projects have been undertaken to develop more efficacious vaccines for prevention, as well as therapy. The third-generation vaccines contain, alongside S-HBsAg, the other envelope proteins of HBV, *i.e.*, middle (M-HBsAg) and/or large (L-HBsAg) surface antigens with their characteristic strongly immunogenic domains preS1 and/or preS2 [9]. Preparations containing two or all three HBV surface antigens exhibited an enhanced immunogenicity [10] and have been introduced as commercial vaccines, e.g., GenHevac, Hepacare^®^, and Bio-Hep-B™, *etc.* [11,12]. However, expression of completely native M- and L-HBsAg in microorganisms, albeit possible [13,14], is still not regularly employed. These antigens are usually produced in special yeast systems [15] or in Chinese hamster ovary (CHO) and other costly mammalian cell expression systems [16]. Therefore, third-generation vaccines are still not commonly administered, except for to non-responders to classical vaccines and other special cases. M- and L-HBsAg have also been considered, together with the core antigen of HBV (HBcAg), as components of postulated therapeutic vaccines for chronic carriers [17,18]. Even if, in contrast to M/L-HBsAg, HBcAg is routinely produced in microorganisms, by itself or carrying fused preS domains [19], the potential therapeutic vaccine based on HBcAg is still undergoing research. On the whole, the alarming HBV prevalence—the number of new infection cases reached *ca.* 250,000 per year in the late 1980s—deficiencies in vaccination programs and emerging problems with immunization efficacy have stimulated research on a new type of effective, but inexpensive and commonly available, vaccine.

Plant-based vaccines seemed an excellent tool for mass prevention. Orally administered plant-associated antigens were reckoned as alternatives, or at least supplements for, injection vaccines derived from yeast or mammalian cells [20,21]. Other premises also indicated that plants could be a potent source of vaccines. Outlays of vaccine production in plant-based expression systems were assumed to be comparable with microbial bioreactors and much lower than in mammalian cells. What is more, in contrast to microorganisms, especially bacteria, it was known that plants expressed eukaryotic proteins in properly folded, modified, assembled and, consequently, native and biologically active forms. Vaccines from plants were considered also advantageous in terms of safety, as naturally free of microbial toxins and pyrogens or human and animal pathogens, including proviruses. However, oral immunization was thought to be the largest benefit and, in the most enthusiastic plans, plant-based vaccines were to be used as edible vaccines. Although initial plans for that idea may have been overly optimistic, the theory behind it was nonetheless quite reasonable. Some contemporary data [22], as well as the successful oral vaccinations, e.g., against polio [23], showed that antigens, particularly assembled into VLPs [24], could overcome the intestinal mucosa barrier and stimulate the immune system of the alimentary tract, gut-associated lymphoid tissue (GALT)—followed by the eliciting of local and systemic immune response [25]. The oral route of vaccine delivery implied two essential advantages, *i.e.*, elimination of complex material processing, mainly purification, and a needle-free administration providing for a minimization of medical service or even facilitating self-application. Additionally, plant vaccines were postulated to be simple in the logistic sense. They were to be produced locally or distributed without the cold chain—as tubers, grains, or dried fruits, which can be stored and transported at ambient temperature. Generally, plant-based vaccines, especially oral ones, were assumed to be cheap and easy, both in production, distribution and application, particularly for developing countries. A leading project of that idea soon became a vaccine against HepB.

## 2. Production of HBV Antigens in Plant Systems

The first-ever plant expression of an antigen of importance for human vaccination [26], its purification and use for injection [27], as well as for oral immunization [28], was reported for the small surface antigen of HBV. In the last 20 years, many projects on plant production of HBV antigens have been realized. Different factors and approaches were tried to increased production scale, as expression systems—stable or transient, plant hosts, promoters, other regulatory and signal sequences, modification of an antigen coding sequence, *etc.*

Stable expression in transgenic plants obtained via *Agrobacterium*-mediated transformation was the main system used for the expression of HBV antigens. The *npt* II-encoding neomycin phosphotransferase was commonly used as a marker gene, but also in single cases, it was either *hpt* or *bar* that determined resistance to hygromycin [29] or herbicide glufosinate [30–33], respectively. The latter can be considered as beneficial to potential oral vaccines and conforming to GMO regulations regarding health.

### 2.1. Small Hepatitis B Surface Antigen

The small surface antigen as the basic HBV immunogen was the main object of research. S-HBsAg was produced in hosts to be exploited for protein synthesis, usually in plants such as tobacco (*Nicotiana tabacum*) [26,34–36], potato [37–44], tomato [34,45–47] or tissue and cell cultures derived from the aforementioned species [42,48–53], in addition to carrot [29] and soybean [51,54]. Other species were also tested, and were as varied as macroalga *Laminaria* [55], banana [48,56], peanut [57], lupin [28,58], lettuce [28,30,59,60] or maize [32,33]. Gene expression was mostly controlled by constitutive promoters—a regular CaMV 35S promoter [26,28,30,37,39,41,46], the 35S promoter with a dual enhancer [26,34,35,38,40,42,44,61,62], and also ubiquitin [36,47–50,54,56] or a hybrid promoter ocs-mas [51]. The tuber- [38,40] seed- [32,33] or fruit-specific [36,47,49,50,53,56], as well as auxin-inducible promoters [29], were tried among non-constitutive promoters. Besides commonly utilized NOS terminator, polyadenylation signals from plant genes were also used [40–42,52,61,62]. Gene expression was also intensified by viral transcription activators, as TEV- or AlMV-5′UTR [26,40–42,44,52,61,62] or codon usage of the S-HBsAg coding sequence was altered according to a plant pattern [42]. Enhanced stability of synthesized S-HBsAg by conjugation with targeting sequences such as the ER-retention signal [36,40,42,47–49,52–54,56] or storage protein signals [32,33,40,42,52], were investigated, as well. Yet, S-HBsAg production in plants did not correlate with used host or vector. Antigen content in plants usually ranged from 0.01 to several micrograms per g FW or tens of hundreds ng/mg TSP [26,28,29,34–39,44–47,52–60], rarely ≥ 10 μg/g FW [30,32,33,40–42,48–51]. It cannot be excluded that high mRNA levels following intensive transcription of expression cassettes has led to gene silencing, instead of efficient translation. Thus, it may be presumed that, apart from active promoters and other cassette elements, a positive effect for S-HBsAg plant expression was found for the general metabolism type and cell substructure, including membrane system and its capacity to deposit foreign VLP-assembled proteins with a high affinity to membrane lipids, similarly to other expression systems or HBV infected cells [9]. For instance, relatively higher S-HBsAg contents were observed in suspension cultures of intensive metabolism, where more of the antigen could be secreted [49], or in mesophyll or parenchymatic cells of tubers or grain in comparison to “compact” cells of tobacco seeds [36]. Recent outcomes show, however, that transgenic plants can produce high yields of S-HBsAg. Lettuce containing a simple expressing cassette composed of the regular 35S promoter and unmodified coding sequence produced S-HBsAg in leaves at mean level of 20 μg/g FW and max. 60 μg/g FW [30]. In another approach, the application of a multiplied seed-specific globulin promoter, together with signal sequences coming from storage proteins, resulted in the accumulation of S-HBsAg in maize seeds at levels up to 71 μg/g [32,33]. Such a production scale, albeit promising and outstanding for transgenic plants, still remains approximately 4–6 times lower than the best results obtained in plants transiently expressing that antigen.

A transient expression system requires repetition of production cycles and a more complex facility, but it renders it possible to avoid problems with obtaining transgenic plants, marker genes and gene silencing, while providing a rapid and robust production of a protein of interest. However, in the case of S-HBsAg, the original results were rather modest. The antigen was expressed in *Nicotiana benthamiana* or tomato, respectively at level of *ca.* ten ng/mg TSP [61,62] or 60–490 ng/g DW [47], which approximately corresponded to several μg/g FW. Probably, the observed average efficiency was an effect of the applied method—tissue infiltration with suspension of *Agrobacterium* carrying optimized vectors (see above) but typical of stable transformation. Only when one of the most potent methods of protein expression based on plant viral vectors, *i.e.*, the MagnICON^®^ system [63], had been exploited, the S-HBsAg level reached 295 μg/g FW [64]. Unfortunately, although this result is the best obtained hitherto, it remains an exception.

### 2.2. Middle and Large Hepatitis B Surface Antigens

Production of other HBV surface antigens, M-HBsAg and L-HBsAg, has been much less studied than that of S-HBsAg. The middle surface antigen was expressed in transgenic potato [37,44], tomato [65,66] and transiently in *N. benthamiana* [61,62], while the large surface antigen was expressed in tomato [67], and both antigens in tobacco and lettuce [31]. Similar to S-HBsAg, different expression cassettes were examined, both simple ones containing the regular 35S promoter and an unmodified antigen coding sequence [31,37,65], and optimized ones including enhanced or organ-specific promoters [44,67], viral transcription activators [44,61,62] or signal peptides [61,62,66,67]. Production efficiency of M- and L-HBsAg varied from 10 to 100 ng/mg TSP or 0.4–2 μg/g FW [37,44,61,62,65–67], exceptionally reaching ≥ 10 μg/g FW [31]. In general, accumulation of M/L-HBsAg was lower than that of S-HBsAg, probably as a result of a decreased ability to form stable VLPs [9,68] as a consequence of additional *N*-terminal preS domains making spatial obstacles [69,70]. An interesting approach to the production of these highly immunogenic determinants is provided by their fusion to S-HBsAg as a carrier. Gene encoding joined fragments of preS1 and truncated S-HBsAg, under control of the glutelin promoter, was expressed in rice seeds [71]. The fusion antigen assembled into VLPs, although its total yield was relatively low—*ca*. 30 ng/g DW.

### 2.3. Hepatitis B Core Antigen

The core antigen of HBV seemed to be ideal for expression in plants. HBcAg spontaneously assembles into durable Capsid-Like Particles [72], which, apart from their own potential application for therapy of chronic HepB [18], can serve as a superb carrier of varied epitopes [73], including those from hepatotropic viruses [19,74]. Although the first report on the relatively efficient HBcAg expression (24 μg/g FW) in transgenic tobacco was published as early as in 1998 [75], for years, efforts devoted to the production of an anti-HBV vaccine have been focused on HBsAg. In the last few years, however, HBcAg has been abundantly produced using transient expression systems, mediated by conventional MagnICON^®^ [76] or novel viral vectors based on PVX, CPMV or BeYDV [77–79]. HBcAg content in *N. benthamiana* was as high as 0.5–2 mg/g FW [76–79], yet it was as low as 10 μg/g FW in cowpea [77].

### 2.4. General Characterization of Plant-Produced HBV Antigens

Plant-produced HBV antigens preserved their physiochemical properties and antigenicity. Density and size of the antigens synthesized in plant cells were correct, comparable to the antigens from human plasma or recombined in yeast [26,30,31,33,38,39,44,49–52,54,56,58,62,64,76,77,79]. Most analyses revealed that glycosylation of HBV antigens did not occur in plant cells, resembling the situation in yeast [33,38,44,50,54,58,64,76,77,79], but putative glycosylated HBsAg proteins, likely following to the plant pattern, were also shown [30,31,51,52]. Most probably, plant-produced HBV antigens were properly folded, as confirmed by ELISA or similar tests using specific antibodies or diagnostic kits designed to detect native antigens [26–67,71,75–79]. Consequently, HBV antigens formed VLPs (in the case of HBcAg, also called CLPs), which were observed directly in plant cells or after purification [26,30,41,51,67,71,75–79]. In summation, numerous experiments have proved that plants can be potent bioreactors producing native HBV antigens, which could be purified for subsequent injection or exploited as oral vaccines.

## 3. Progress and Barriers of Oral Immunization

The keywords “edible vaccines” epitomized the way in which plants bearing vaccine antigens were initially imagined to be applied for. Thus, the oral delivery route and immunization through intestinal mucosa were investigated in many trials, although such a “vaccination by feeding” has caused difficulties since the very beginning of research. A problem of forced ingestion was treated as rather subsidiary, because the real challenge was to establish an effective immunization protocol. Experiments on oral vaccination against HepB were mostly conducted using preparations containing S-HBsAg [28,30,32,40–44], but also M-HBsAg [44,80] and HBcAg [76], and two main methodological approaches were examined.

The first, indeed the predominant one, was high-dose and multiple administration of HBs antigens, usually supplemented with a strong mucosal adjuvant activating GALT [32,40–43,44,80]. Most likely, such an immunization procedure was assumed [21,81] in view of concerns that orally administered HBsAg might be digested in the gut before eliciting any immune reaction. In fact, consecutive (every few days) orally administered raw plant tissue, mainly potato tubers [40–43,44,80] or tissue extract [32,80] bearing high HBsAg doses, from several up to even *ca.* 42 μg/mouse [41] or several hundred μg/human [43], and adjuvanted with CT or CTB [40–42,44,80] or LT [32], elicited a significant immune response, comparable to standard injection vaccination with rHBsAg, and reached max. titer of anti-HBs antibodies in serum 700–5000 mIU/mL [32,40–44,80]. However, a high-dose antigen administration via ingestion was only a step in the full immunization procedure, in which parenteral delivery of rHBsAg was an integral part. All the same, the main finding of those experiments was that at least a single antigen injection appeared to be essential for efficient oral boosting [40–42,80], also in the case when the non-adjuvanted antigen was administered [43,46]. Also, some immune alertness, e.g., anti-HBs antibodies maintained for years after a regular three-dose injection vaccination, was crucial for the efficacy of booster vaccination [43]. Yet in some experiments, when mice were fed with a non-adjuvanted plant-associated HBs antigen, none or low reaction was observed and the subsequently injected vaccine did not substantially improve immune reaction [41,46]. Generally, albeit effective in most cases, such a vaccination manner would be very problematic to put into practice due to the administration of bulky plant tissue, sometimes containing harmful secondary metabolites as in tubers, and it raised controversies concerning the use of such mucosal adjuvants as CT- or LT-derivatives for mass vaccination [82].

Concurrently performed trials were based on exclusively oral administration without any injection. The idea of this research was also to provide feasibility of oral vaccination by minimization of the amount of neutral plant material required for immunization, hence the consequent administration of low-dosed HBsAg, possibly without exogenous adjuvants. Experiments on oral delivery of raw lettuce leaves or lupin callus tissue showed that low doses, *ca.* 0.5–1 μg, of unadjuvanted S-HBsAg, delivered every 1 or 2 months evoked systemic immune response [28,59] However, anti-HBs antibodies were elicited at a level only slightly above the protective minimum, *i.e.*, 10 mIU/mL of serum. The results revealed also that intervals between antigen delivery affect immune response, as the anti-HBs titer in mice fed the callus tissue for five consecutive days was two times lower than in animals gavaged only once. Similarly, the antibody level in volunteers who consumed lettuce at days 0, 7 and 30 did not reach the protective minimum [60]. Regularities of the oral vaccination course and the still onerous application of perishable and bulky raw plant material indicated that a new form of a vaccine had to be applied to conduct immunization trials according to a controlled and convenient regime. Such a preparation should be size-reduced, durable and contain a defined antigen dose, for instance lyophilized tissue and its derivatives. Processed plant tissue containing low doses (100 ng) of S-HBsAg delivered only orally at 1 or 2 months induced systemic response above the anti-HBs’ protective titer [30] and comparable to parenterally-orally administered unadjuvanted antigen [41,46], while high doses were ineffective. Nevertheless, despite that success, observed reactions were too low (max. 20 mIU/mL) for practical use.

Orally administered HBsAg also induced S-IgAs [30,32,80], and their production corresponded with the antigen dosage. Mucosal response in the intestine was considered advantageous as a part of extended protection [83]. An intensive mucosal antibody response is desirable for such antigens as CTB, LT, *etc.* since S-IgAs may prevent infection of pathogens penetrating mucosa [84]. However, in the case of blood-borne pathogens such as HBV, where systemic response is required, S-IgA production in response to an orally administered antigen may be considered as ambiguous. It was observed that S-IgAs were produced simultaneously with oral tolerance acquisition [85].

Oral tolerance is characterized as suppression of immune response to antigens present in the intestinal lumen and has been known as one of the possible effects of GALT activity [86]. This phenomenon, together with similar activities of the Mucosa Associated Lymphoid Tissue (MALT), linked to all mucous membranes of the organism (intestinal, rectal, nasal, bronchopulmonary, *etc.*), plays a vital role as being permanently exposed to innumerable antigens, discriminates pathogens from harmless or neutral antigens and subsequently induces active or suppressive response [25,87]. Yet, in earlier research on plant-based oral vaccines, the role of this barrier was likely either underestimated or not fully realized. In the case of HBV, it even may have been assumed that its antigens assembled into durable VLPs were particularly suitable as oral vaccines, since such nanoparticles, in contrast to soluble proteins, constitute effective immunogenes, also delivered to mucosal membranes [88,89]. Synergistically, VLPs resembling virions could alert the GALT as a “danger” signal of viral infection [24].

Recurring difficulties with oral immunization against HBV and other blood-borne pathogens gradually caused a revision of views on the effectiveness of plant-based vaccines [90–92]. Mechanisms of oral tolerance are still under study. Yet, it is known that depending on conditions of antigen delivery, a local response in the intestinal mucosa may develop into an adaptive systemic immune response or unresponsiveness [93,94] and antigens delivered in high doses and/or exposed frequently or for long duration for to GALT cells are conducive to tolerance [95]. Hence, it is likely that HBV antigens initiated some response at first, but high-dose and/or extended oral immunization led to suppression of response. For instance, the high-dosed antigen in an “edible vaccine” would probably be split into lower undefined sub-doses, repeatedly and for an extended duration exposed to GALT, thus adsorbed as the dietary component [96]. There are no published data on high-dose strictly oral immunization (only parenteral-oral), but probably such an immunization method would not be effective, as shown in similar studies based on relatively frequent antigen ingestion [60]. As a solution of that problem, it was assumed that a controlled “one-shot” immunization using low and strictly defined doses of plant-associated HBsAg in a volume-reduced lyophilized tissue would be effectual. Unfortunately, such a formula also appeared to evoke systemic response only slightly above the minimal protective level [30]. What is more, it may be supposed that any procedure of oral immunization with plant tissue bearing HBsAg would lead to a suppression of immune response. Regulatory T lymphocytes (Treg), crucial mediators of mucosal tolerance development [97], were stimulated by extremely low (0.5–100 ng) HBsAg doses in small amounts (max. 1 mg) of lyophilized tissue. Moreover, plant tissue without any antigen also induced a certain growth of Treg population [85]. Apparently, natural plant components induce tolerance, which then may expand to associated HBsAg and be enhanced in correlation with the antigen dosage. It is also possible that VLPs HBsAg coming from a non-mucosal pathogen were recognized by the GALT as a neutral antigen [24].

Problems with oral immunization could be observed also for HBcAg, which is a naturally strong antigen inducing both humoral and cellular responses [18]. The partially purified, high-dosed (500 μg) and alum-adjuvanted orally delivered antigen induced detectable but not very high response in comparison to placebo, while an intensive production of serum anti-HBc antibodies was elicited only by intranasal immunization [76]. However, the nasal cavity and the intestine are different milieus, in which distinct immune mechanisms are active. Nasal-associated lymphoid tissue (NALT) is a particularly active mucosal immune system, hence most of mucosal vaccines are intranasal [98]. However, intranasal immunization using HBcAg indicates that if not oral, other mucosal membranes can be recipients for vaccines against blood-borne pathogens such as HBV.

All in all, observed problems, regardless of different vaccination protocols and preparations, revealed that a really efficacious plant-based oral vaccine against HBV was much more difficult to create than assumed. On the other hand, knowledge gained in those research projects may be perceived as a hidden, but powerful capital to invest.

## 4. Plant-Produced HBV Antigens as Injection Vaccines

For years, plant-derived anti-HBV injection vaccines have been slightly placed at the sidelines with the focus being on oral vaccines, even though S-HBsAg expressed for the first time in plants [26] was purified and administered intraperitoneally [27]. Although surely more complicated and pricey, parenteral (intramuscular, intraperitoneal or subcutaneous) delivery of HBV antigens had an essential superiority over oral immunization, as appropriate vaccination protocols had been already well-developed and verified. Because only purified antigens can be injected in practical applications, efficient methods of extraction from plant tissue and purification of the HBV antigen have to be first elaborated. Moreover, the highest possible yield of the antigen is required to make this process cost-effective. In the first experiments, HBV antigens designed for purification, mainly S-HBsAg but also M-HBsAg and chimeric preS1-S and HBcAg, were produced using optimized vectors (see above) in transgenic plants, or transiently on a relatively low level—from *ca.* ten ng to several μg/g FW, or ten of hundreds ng/mg TSP [26,34,39,52,61,62,71], exceptionally >20 μg/g FW in the case of HBcAg [75]. Although antigens were suitably purified for injection [26,27,34,51,52,61,62,71], that yield would be unsatisfactory thereafter. S-HBsAg was synthesized at a substantially higher level (74 μg/g FW) in a soybean suspension culture [51], but a real breakthrough came with the utilization of virus-based transient expression systems for robust production of S-HBsAg [64] and HBcAg [76–79] with yields as high as 0.3–2 mg/g FW. Formation of macromolecular VLPs by HBV antigens made it possible to exploit the standard [14,15] and relatively inexpensive technique of protein purification such as ultracentrifugation [27], usually in sucrose [26,39,51,52,61,62,64,71,75,76,79] or cesium chloride gradient [26,56]. These methods were used as either final or preliminary before concentration via ultrafiltration [27,51,61,62,76] or immunoaffinity purification [26,64]. The final efficiency of purification for HBV antigens amounted to 0.4–3 μg/mL [39,51,52,56,61,62] and could be increased up to 100 μg/mL by ultrafiltration [27], *i.e.*, a concentration meeting the standards accepted for commercial vaccines. However, the most significant aspect for vaccination purposes was that plant-derived VLPs had identical properties [26,51,64,75–79] and evoked comparable systemic humoral responses as vaccines obtained in other expression systems. Anti-HBs antibody titers reached up to 800 mIU/mL when S-HBsAg was injected [27,52,64], or 1165 mIU/mL in the case of M-HBsAg [62] and for HBcAg it was the same antibody titer as the antigen recombined in *E. coli* [76]. Those results strongly confirm the equivalence of plant-derived and classical recombined anti-HBV injection vaccines.

## 5. Possible Scenarios for Plant-Based Anti-HBV Vaccines

Despite the 20 years of research, practical utilization of plant-based anti-HBV vaccines appears to be more problematic than had been expected. Reports of postulated actual clinical trials [21,81,99,100] have not been published to date, not to mention vaccine registration or commercial scale production. In the meantime, recombined anti-HBV vaccines provided substantial prophylaxis and control of HepB [101,102] due to the considerably reduced price per dose (in various countries *ca.* $1–20), increased availability and mass immunization programs [103]. Anti-viral HepB therapy schemes using IFN-α or lamivudine and other drugs have also been essentially developed [104]. Consequently, the number of HBV chronic carriers dropped in North and South America (except for some endemic areas), Australia and Western Europe to <1% of the population, and exposure rate below 2%, while in Central and Eastern Europe, the Mediterranean region, the Middle East, the Indian subcontinent and Japan, prevalence declined to intermediate 1%–7% and the risk of infection to 10%–60% [105,106]. Progress in HepB prevention and therapy using classical vaccines and treatments might call into question reasons for the introduction of plant-based vaccines.

However, such a point of view could be named “conservative” and unjust. Present epidemiological data on HepB, as well as scientific and technological progress of biopharming, provide a solid rationale for further research on plant-based vaccines against HBV. One third of the global population still lives in areas with a high risk of HBV infection. In the sub-Saharan Africa, Central and South-Eastern Asia and China, 70%–90% of the population is exposed to the virus, and prevalence reaches ≥ 8%. The number of chronic carriers worldwide is slowly, but steadily, growing [105,106], and it has increased from 250 to 300 million at the moment of the introduction of anti-HBV recombinant vaccines up to a current 370 million. As it is estimated, 600,000–1 million people die every year from HepB or post-disease complications, such as cirrhosis or HCC [106,107]. Vaccines against HBV are presently relatively cheap, but problems with their distribution (e.g., the cold chain), deficient infrastructure, and a lack of highly qualified medical personnel result in delayed or insufficient national programs of HepB prevention [102,105–107]. Anti-viral HepB therapy, although it has essentially progressed, remains an economical barrier for inhabitants of the most threatened poorer countries [104]. Therefore, easily implementable methods of anti-HepB prevention continue to be necessary. Most of prerequisites of plant-based vaccines keep topicality, albeit originally thought as “edible vaccines” turned out to be unusable. Plant-derived formulations, both based on purified antigens and partially processed material and administered by injection or orally, may still be considered as alternative or auxiliary to standard anti-HBV vaccines, since they have the potential for cost-effective production and processing and/or simplified vaccination [99,108,109].

Vaccines obtained using transient systems look the most promising. Protein expression based on virus-derived vectors is a predominant trend in today’s and probably future pharmaceutical plant biotechnology [110]. Milligram yields of S-HBsAg or HBcAg [64,76–79] provide a solid basis for efficient processing of plant material on a semi- or technical scale. Purification of HBV antigens has also been developed and needs only to be upgraded. An obvious question remains of whether plants may be competitive to other expression systems. Nowadays, the yield of transient expression makes up for only 1/20–1/25 of yeast production [15]. After that, however, it is economically comparable and the plant production scale can still be increased [111]. Although synthesis of “humanized” proteins in yeast has already been elaborated [112], plants also offer engineered glycosylation pathway [113], so plant-derived products would be completely native. It is almost certain that plant systems producing HBV antigens would be much more cost-effective in comparison to mammalian cells [111]. Regardless, some issues of plant-derived injection vaccines still require elaboration or optimization, e.g., repeatability of high antigen expression, M/L-HBsAg synthesis or stability in long-term storage. Distribution of a vaccine in a durable lyophilized form [114], suspended directly before injection, is worth considering. First of all though, plant-produced HBV antigens delivered parenterally were equally immunogenic as classical vaccines [27,52,62,64,76]. Research on plant-derived anti-HBV injection vaccines arrived at a point of clinical trials as a logical consequence. It would seem that nothing is standing in their way so they are expected to appear in the near future. In short, plant-derived anti-HBV injection vaccines have promising prospects as equivalent if not more profitable than those obtained in traditional systems.

On the other hand, it can be assumed that HBV antigens obtained via transient systems and delivered parenterally are generic vaccines in effect and may entail higher costs due to required complex production facilities, cold chain distribution and additional delivery equipment. Thus they do not solve issues of universal access and simplified distribution and vaccination. As it was proposed previously [100], purified HBs antigens may likely be used as oral or generally mucosal vaccines. Orally administered purified HBsAg and adjuvanted with various substances, elicited humoral response [115–117], similar to intranasally delivered HBcAg [76]. However, such a manner of anti-HBV vaccine delivery would rather be supplementary. It would also require additional equipment (applicators, *etc.*) and cold chain, not to mention further studies on the immunization procedure, while parenteral injection is well known and verified.

**Figure 1:**
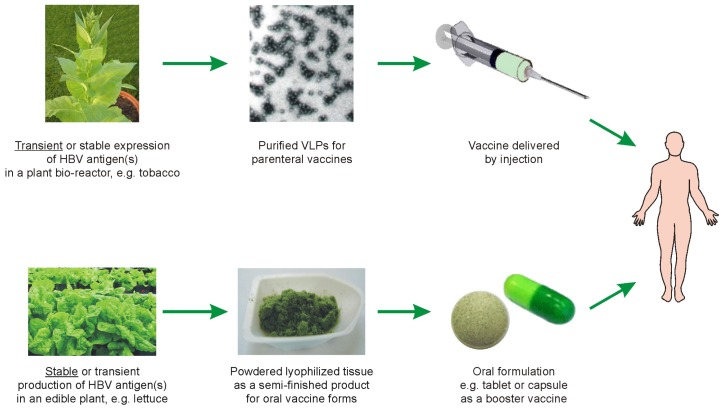
Potential plant-derived parenteral and oral vaccines for immunization against Hepatitis B Virus. Note: underlined—mostly exploited today or more probable antigen production systems for particular vaccine types.

Plant-based oral vaccines can yet be considered as meeting half-way the original concept of cheap and commonly available vaccines against HepB. It cannot be denied, though, that the slogan “edible vaccines” has become utterly obsolete, and general interest in plant-based anti-HBV oral vaccines has diminished. Moreover, so far, data indicate that efficacious solely oral immunization is possible against mucosal pathogens, but is very difficult in the case of blood-borne ones. More advanced methodologies of vaccination against HBV were also either impractical such as e.g., a combined parenteral-oral antigen delivery with CTB-adjuvanted raw plant tissue or only minimally effective as solely oral administration of the antigen in lyophilized tissue. However, based on previous trials, a novel vaccine may be proposed, which unifies advantages of both aforementioned methods (Figure 1). According to this combined approach, a partially processed plant tissue carrying HBV antigen(s) may be applied as a component of injection-oral vaccine and generally as a booster vaccine. A parenterally delivered antigen at priming usually elicits a low humoral response, but makes GALT susceptible and recipient to the oral booster with plant-associated HBsAg [40–44,80]. Consequently, a barrier of oral tolerance is probably avoided or reduced, thus facilitating successful oral boosting. Effective boosting of previously vaccinated subjects via ingestion with raw plant tissue was shown [43]. However, to conform to medical and practical requirements, plant tissue should be converted via lyophilization [30,31] or extraction using organic solvents [32,33]. As shown recently, such a coarsely processed and LT-adjuvanted tissue containing high-dosed S-HBsAg can be effectively used in the second step of injection-oral immunization procedure, when it elicited up to 4600 mIU/mL of anti-HBs antibodies [32]. A low-dose and non-adjuvanted immunization regime appeared to be also sufficient as it induced *ca.* 800 mIU/mL of anti-HBs, comparable to three doses of the injected antigen [118]. Hence, it is very likely that the processed tissue carrying antigen(s) may be used by itself as a periodically administered anti-HBV booster vaccine.

Although promising, the parameters of lyophilization or extraction of plant material need to be considerably upgraded to improve antigen stability. A significant loss of HBsAg proteins was observed during the processing and storage of prepared material. As much as 90% of VLPs formed by S-HBsAg were degraded during freeze-drying, but the antigen was later stable when stored [30]. For M- and L-HBsAg, especially preS domains and VLPs were susceptible to alteration or degradation [31]. In comparison, extraction using organic solvents was not so dramatic, but after one week of storage 25%–45% of S-HBsAg was lost [33]. There are no data on lyophilization or rough extraction of plant-associated HBcAg at the moment. Processing yield can be increased by manipulation of physical conditions, optimization of extraction mixtures or addition of lyoprotectants, *etc.* Nevertheless, tissue processing makes it possible to concentrate and standardize antigen doses and it would facilitate a controlled immunization regime using a size-reduced, durable and convenient vaccine form. Such a form can be tablets [30] or capsules or simply portioned powdered tissue. An oral formula provides also opportunity to adjust its chemical composition to be optimal for immunogenicity. Immunomodulators, e.g., interleukins or adjuvants as CpG oligos, chitosan, bile salts and other chemicals of lipid nature [116,117,119], can be added exogenously during vaccine formulation. Moreover, some commonly occurring plant components, such as vitamins, carotenoids, oils, *etc.*, as well as some secondary metabolites, e.g., terpenoids, saponins or lectins, *etc.*, have adjuvant or immunomodulatory properties [120–122]. To summarize, purely oral anti-HBV vaccines appeared to be an unrealistic dream, but recent outcomes provide a rationale for further research on the oral formula as a novel, plant-derived booster vaccine against hepatitis B.

## 6. Conclusions

Although twenty years of research did not result in a real plant-based vaccine against HepB, essential scientific and technological progress has been made. Efficient stable and transient plant expression systems have been developed for the production of each HBV antigen, in addition to improved methods of plant tissue processing and antigen purification, as well as new effective immunization procedures. Plant-produced HBV antigens can be used as injection and mucosal vaccines, including oral vaccines in a tablet or capsule form prepared from coarsely processed material. Purified injected antigen(s) can be used as a regular vaccine, oral formulations as a booster vaccine, while both vaccine types may be combined in the parenteral-oral immunization procedure (Figure 1). The most promising prospects seem to be associated with injection vaccines, characterized by robust transient production, developed purification methods and proved immunogenicity. However, their use may involve expenses connected with production technology, cold distribution and delivery equipment. The oral formulation could be based on only minimally processed, *i.e.*, lyophilized or extracted plant material. Yet, their utilization only as a booster vaccine and the necessary association with injection may require some organizational arrangements. In any case, to be promising, plant-derived anti-HBV vaccines need to be improved and their cost has to be reduced. Hence, some optimization research is recommended, such as: (1) stability and repeatability of scaled-up production of HBV antigens; (2) increased efficiency of downstream antigen purification and following processing for injection vaccines or plant tissue conversion for oral ones; (3) preparation of durable vaccines, with long shelf-life, optimally at ambient temperature; (4) for oral vaccines—selection of an appropriate mucosal adjuvant; (5) a verified effective regime of oral booster repeated vaccinations; and, finally; (6) elaboration of definite formulas of vaccines. Those realistic expectations seem to be either a necessary precondition or equally important as pre-clinical and clinical trials.

Production of HBV antigens in bioengineered plants and their processing, according to regulations of good manufacture practice (GMP), as well as their proved efficacy, biosafety and/or bioequivalence *etc.*, are indispensable to approved vaccines. Plant-derived vaccines based on purified VLPs and delivered by injection would probably have the advantage over oral formulations. It can be expected that the first ones could be produced under GMP before long, similar to other vaccines [123]. Furthermore, as generics, they would have somewhat preferential position in clinical trials. Oral anti-HBV vaccines, even optimized, would be a new type of a vaccine, though, and thus they would entail meticulous clinical research. Therefore, when verified, both plant-based vaccines against HepB may by admitted by health agencies, but it would be expected much sooner for injection than for oral formulations. However, positive decisions of FDA, EMA and analogous agencies would be absolutely relevant for subsequent commercialization and introduction of plant-based anti-HBV vaccines into practice.

Truly efficacious and reasonably priced plant-based vaccines against HepB are attainable and still advisable. Plant-derived injection vaccines can at least partially replace present vaccines. An oral formulation which would cover periodic booster vaccinations, if not substitute two out of the three doses of the primary vaccination, could be seen as a worthwhile alternative. All in all, plant-based vaccines can help to alleviate the problem of persisting HepB and offer a good strategy for health improvement in many countries worldwide.

## Abbreviations

AlMV: Alfalfa Mosaic Virus
BeYDV: Bean Yellow Dwarf Virus
CaMV 35S promoter: promoter of 35S RNA of Cauliflower Mosaic Virus
CHO: cell line derived from Chinese hamster ovary
CLPs: Capsid-Like Particles
CPMV: Cowpea Mosaic Virus
CT: Cholera toxin
CTB: Cholera toxin subunit B
DW: dry weight
EMA: European Medicines Agency
ER: endoplasmic reticulum
FDA: U.S. Food and Drug Administration
FW: fresh weight
GALT: Gut-Associated Lymphoid Tissue
GMP: Good Manufacture Practice
HepB: Hepatitis B
HBV: Hepatitis B Virus
HBcAg: Hepatitis B core Antigen
HBsAg = HBs antigen(s): (any) Hepatitis B surface Antigen(s)
rHBsAg: recombinant Hepatitis B surface Antigen
S-, M-, L-HBsAg: small, medium or large HBsAg
HCC: Hepatocellular carcinoma
LT: Heat-labile enterotoxin
MALT: Mucosa-Associated Lymphoid Tissue
mIU/mL: milli-International Unit/mL = unit of titer of anti-HBs antibodies
NALT: Nasal-Associated Lymphoid Tissue
NOS: nopaline synthase
PVX: Potato Virus X
S-IgA: secretory IgA
SVP: subviral particle
TEV: Tobacco Etch Virus
TSP: total soluble protein
5′-UTR: 5′-untranslated region of mRNA
VLPs: Virus-Like Particles

## Supplementary Files

Supplementary File 1 Supplementary Material (PNG, 290 KB)
