# Supplementary figures and images for: The Twenty-Year Story of a Plant-Based Vaccine Against Hepatitis B: Stagnation or Promising Prospects?
Source: Int J Mol Sci. 2013 Jan 21;14(1):1978–98. doi: 10.3390/ijms14011978 (PMC3565360; doi:10.3390/ijms14011978)

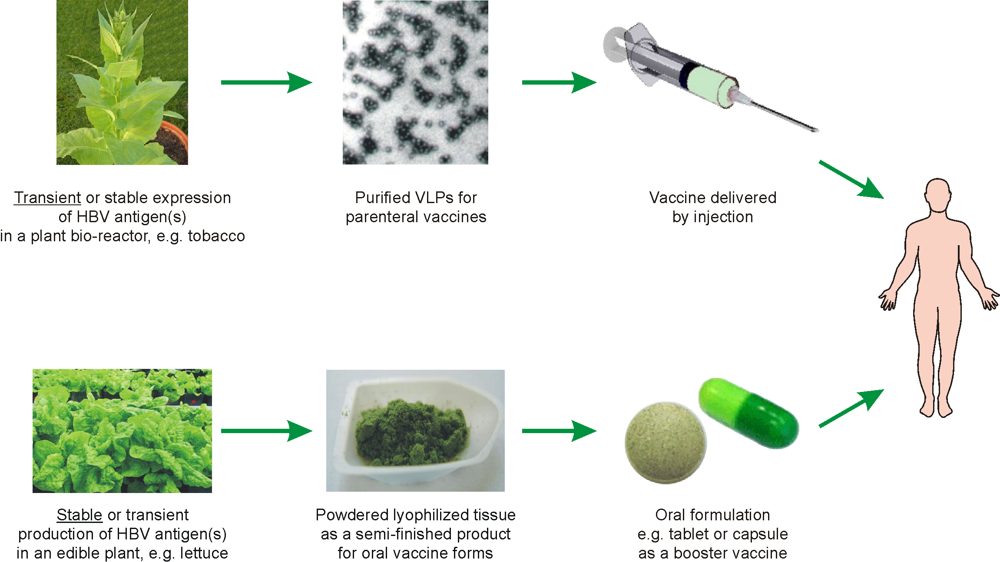

Supplement: Supplementary File 1 — Supplementary Material (PNG, 290 KB) [file ijms-14-01978-s001.png]
